# Immunization with H1, HASPB1 and MML *Leishmania* proteins in a vaccine trial against experimental canine leishmaniasis

**DOI:** 10.1016/j.vaccine.2007.05.010

**Published:** 2007-07-20

**Authors:** J. Moreno, J. Nieto, S. Masina, C. Cañavate, I. Cruz, C. Chicharro, E. Carrillo, S. Napp, C. Reymond, P.M. Kaye, D.F. Smith, N. Fasel, J. Alvar

**Affiliations:** a WHO Collaborating Centre for Leishmaniasis, Centro Nacional de Microbiología, Instituto de Salud Carlos III, Majadahonda, Spain; b Department of Biochemistry, University of Lausanne, Epalinges, Switzerland; c RMF Dictagene, Lausanne, Switzerland; d Immunology and Infection Unit, Hull York Medical School and Department of Biology, University of York, Heslington, York, United Kingdom

**Keywords:** Vaccine, Leishmaniasis, Canine

## Abstract

The protective capabilities of three *Leishmania* recombinant proteins – histone 1 (H1) and hydrophilic acylated surface protein B1 (HASPB1) immunized singly, or together as a protein cocktail vaccine with Montanide™, and the polyprotein MML immunized with MPL^®^-SE adjuvant – were assessed in beagle dogs. Clinical examination of the dogs was carried out periodically under blinded conditions and the condition of the dogs defined as asymptomatic or symptomatic. At the end of the trial, we were able to confirm that following infection with *L. infantum* promastigotes, five out of eight dogs immunized with H1 Montanide™, and four out of eight dogs immunized with either the combination of HASPB1 with Montanide™ or the combination of H1 + HASPB1 with Montanide™, remained free of clinical signs, compared with two out of seven dogs immunized with the polyprotein MML and adjuvant MPL^®^-SE, and two out of eight dogs in the control group. The results demonstrate that HASPB1 and H1 antigens in combination with Montanide™ were able to induce partial protection against canine leishmaniasis, even under extreme experimental challenge conditions.

## 1 Introduction

*Leishmania* are protozoan parasites that cause a wide spectrum of human diseases from self-limiting cutaneous leishmaniasis to potentially fatal visceral infection. Zoonotic visceral leishmaniasis (ZVL) caused by *Leishmania infantum* is an emerging veterinary and public health problem in endemic areas of the Mediterranean basin, extending to the middle East, Asia and South America (*L. chagasi*) [1,2]. Transmission between dogs, or from dogs to man occurs by the bite of a phlebotomine sand fly. Pet dogs are the principle reservoir for maintaining the domestic cycle of parasites whilst stray dogs and wild canids maintain the peridomestic cycle and appear to spread the disease [3]. Epidemiological surveys have demonstrated high infection rates of dogs in endemic areas (67% in Majorca, Spain), even though *Leishmania* infection remains subclinical in most cases [4,5].

Current strategies to control ZVL are essentially ineffective. The treatment of dogs with drugs such as antimonials or amphotericin B has a high cost and low efficacy, with relapses occurring in the majority of dogs. A significant proportion of these dogs, although clinically asymptomatic, are also able to transmit parasites to the sand fly [6,7]. Furthermore, successive treatment following relapse could introduce resistant strains of parasites, thus representing a clear risk to human health [8]. The mass culling of infected dogs has had mixed results in reducing human leishmaniasis prevalence in endemic areas and is generally not accepted for ethical and social reasons [9–12]. Therefore, the development of a protective vaccine in dogs would be an important tool to efficiently control canine visceral leishmaniasis (CVL) thus reducing the chances of infectivity to sand fly vectors and consequently the transmission to humans.

In recent years, efforts have been made by several different groups to develop vaccines against canine leishmaniasis. Killed *Leishmania* antigen plus bacillus Calmette-Guérin (BCG) adjuvant [13] were used in phase I and II clinical trials in Brazil with high protection rates, however, this formulation failed to detect any significant differences between vaccine and placebo groups in phase III field assays [14]. The glycoprotein enriched fucose mannose ligand (FML) vaccine of *L. donovani* in combination with QuilA adjuvant, was shown to elicit a protective effect in the field [15] and to further block transmission by keeping the vaccinated dogs free of parasites [16]. More recently, an experimental vaccine trial using *L. infantum* antigen proteins excreted – secreted from promastigotes (LiEASAP), together with muramyl dipeptide (MDP) adjuvant, was successful in preventing *L. infantum* infection [17]. The use of a more defined *Leishmania* antigen as vaccine candidate included such preparations as the recombinant multi-component antigenic protein, named Q, which when formulated with BCG led to 90% protection in immunized dogs under experimental infection conditions. However, the absence of an adjuvant control group in this study undermined the significance of antigen specific protection [18]. Defined antigens in the form of DNA have also been trialed with some success [19,20]. In the latter study, a cocktail consisting of cysteine proteinase type I (CPB) and type II (CPA) antigens from *L. infantum* were used in a heterologous prime-boost (DNA-protein) vaccination against experimental canine leishmaniasis. However, vaccination with a recombinant *L.infantum* CPA and CPB preparation using canine IL-12 as adjuvant did not protect dogs from infectious challenge [21]. The first defined recombinant vaccine antigen to undergo phase III field assays was recently described [22]. The antigen used was the polyprotein MML, also known as Leish111f [23,24]. This antigen when used in combination with either MPL^®^-SE or Adjuprime adjuvants failed to protect dogs from natural *Leishmania* infection or disease progression.

In this work, we examined the protective capability of the recombinant histone H1 (H1) and hydrophilic acylated surface protein B1 (HASPB1) as novel antigens in a vaccine against experimental canine leishmaniais. Both H1 and HASPB1 have previously been shown to be protective in the mouse [25,26] and for H1, in a monkey model [27] of leishmaniasis. We therefore examined the immunogenicity and efficacy of *L. infantum* H1 and *L. donovani* HASPB1 antigens in combination with Montanide™ adjuvant singly, or together as a protein cocktail vaccine, in dogs under high dose experimental challenge conditions. In addition, the previously examined MML polyprotein [22] in combination with MPL^®^-SE adjuvant was included in this trial. Clinical, parasitological and immunological examination of the animals were carried out for a period of 64 weeks following infection.

## 2 Materials and methods

### 2.1 Parasites

The *L. infantum* strain JPC (MCAN/ES/98/LLM-722) was isolated from the spleen of a dog with patent canine leishmaniasis. Parasites were grown in NNN medium for 2 weeks and sub-cultured to complete RPMI-medium (RPMI 1640, Gibco, Paisley, UK) supplemented with 100 UI/ml of penicillin, 100 μg/ml of streptomycin, 2 mM l-glutamine, 5 × 10^−5^ M 2-mercaptoethanol and 10% heat inactivated foetal calf serum (Biological Industries, Israel). Parasites were further cultured in acidified complete RPMI-medium (pH 5.5 at 27 °C) for 3 days to promote metacyclogenesis. The virulence and infectiveness of this strain has been confirmed in dogs by others [28].

### 2.2 Vaccine antigens

The *L. infantum* histone H1 (DQ232891) was cloned into the pGEX-KG vector (Amersham Biosciences), expressed in *Escherichia coli* and purified using GST affinity resin (Amersham Biosciences), as previously described [25]. HASPB1 (AJ011810) was cloned into the pET15b vector, expressed in *E. coli* and initially purified on a Ni-NTA resin then further purified using an Anion exchange column (Amersham Biosciences). The histone H1 and HASPB1 proteins were purified from endotoxins under pyrogenic free conditions in 1× PBS on a Superose 12 column (Amersham Biosciences). Endotoxin levels were determined using a Chromagenic LAL kit (Bio-Whitttaker) and ensured to be below 5 EU/mg. The final concentration and purity of proteins was determined by SDS-PAGE, RP-HPLC (Waters Alliance) and MALDI-TOF mass spectrometry (Perkin-Elmer Biosystems). One milligram samples were lyophilized in 1× PBS + 5% mannitol for histone H1. HASPB1 was prepared as 1 mg/ml aliquots in 1× PBS. The polyprotein MML was prepared by Novartis Animal Vaccines Ltd. (Braintree, UK) following previously reported procedures [24].

### 2.3 Animals

Forty eight beagle dogs were used for this study. Animals between 8 months and 3 years old were purchased from dog breeders in different regions of Spain. All dogs had received routine vaccinations. Absence of *Leishmania* infection was confirmed prior to commencement of the study in all the animals by lack of specific serum antibodies to *L. infantum* as measured by indirect immunofluorescent antibody test (IFAT) as previously described [7] and by ELISA. Culture and PCR analysis of different tissues (skin, peripheral blood, bone marrow, and popliteal lymph node) were also negative in all cases and specific lymphoproliferative response to leishmanial antigens was not detected following *in vitro* culture of peripheral blood mononuclear cells (PBMCs). Dogs were kept in our own facilities at the National Centre for Microbiology, Majadahonda (Madrid), under constant veterinary care.

### 2.4 Immunization and experimental infection

Dogs were distributed into seven groups (eight animals per vaccine and control groups and four animals per adjuvant group) taking into consideration sex, weight and age. Sex ratio (m/f) was 4/4 or 2/2 in all groups except for one group where it was 1/3. Mean age of the animals for each group varied between 14.7 and 18 months, and the mean weight of the animals for each group ranged from 9.5 to 11.7 kg. No statistically significant differences exist between the groups due to mean age or weight (*p* < 0.05).

Dogs received three intradermal doses (dorsum; 1 ml/dose) of each vaccine formulation for a period of 3 months. On day 0, dogs from group HASPB1 and group H1 received 100 μg of HASPB1 or histone H1 protein. On days 30 and 60 the dogs received 45 μg of either protein. Dogs in group HASPB1 + H1 received a cocktail of histone H1 and HASPB1 (100 μg each) at day 0, and 45 μg of each protein on days 30 and 60. The adjuvant used for dogs in groups HASPB1, H1 and HASPB1 + H1 was Montanide™ ISA 720 (70% formulation, according to manufacturer's instructions, SEPPIC), given on days 0 and 30. The final immunization on day 60 for groups HASPB1, H1, and HASPB1 + H1 was prepared in the absence of adjuvant to avoid side effects observed following the second dose. These dogs were thus immunized with proteins formulated in 1× PBS for the third vaccination. Animals from group Montanide were inoculated with Montanide™ adjuvant only on days 0 and 30. On day 60 these dogs were inoculated with 1 ml of PBS. Dogs from group MML received three doses of 45 μg of MML plus a 50 μg/dose of MPL^®^-SE adjuvant on days 0, 30 and 60. Animals from group MPL-SE received the MPL^®^-SE adjuvant preparation only. Dogs in the positive infection control group received three doses of 1 ml PBS on days 0, 30 and 60. Forty-five days following the final immunization, all dogs were infected intravenously with 1 × 10^8^ virulent *L. infantum* promastigotes.

### 2.5 Clinical examination and laboratory analysis

Routine clinical and laboratory evaluation of the animals was carried out every 4 weeks for a total of 64 weeks. In each evaluation dogs were weighed and their general health status was examined. Biological samples were obtained for laboratory analysis as described below.

#### 2.5.1 Clinical examination for symptoms of CVL

Examination of the dogs for endpoint determination of CVL was carried out blind by an independent clinical veterinarian at weeks 16, 34, 42, 52, 57 and 62 post-challenge. The condition of the dogs was defined according to the external signs of canine leishmaniasis as asymptomatic (no external sign of canine leishmaniasis), or symptomatic when the dogs showed one or more external clinical signs of CVL including, lymphadenopathy, onychogryphosis, alopecia, cutaneous lesions, weight loss or keratoconjunctivitis.

#### 2.5.2 Haematology and biochemistry

Blood samples taken from the jugular vein were kept in Ca^2+^-EDTA tubes and were analyzed for hematocrit, total erythrocytes, leukocytes, lymphocytes, and platelet counts by an automated blood cell counter (Vet ABC, Scil, France). Serum levels of alanine aminotransferase (ALT), aspartate aminotransferase (AST), blood urea nitrogen (BUN), creatinine, alkaline phosphatase (alk. phosph.), globlulines and total proteins were determined by a biochemistry serum analyzer (IDDEX, Netherlands).

#### 2.5.3 ELISA

Serum levels of specific antibodies to *L. infantum* SLA [29], rK39 [30], HASPB1, histone H1 or MML were analyzed by ELISA. Microtiter plate wells were coated with either SLA (1 μg), rK39 (50 ng), HASPB1 (50 ng), histone H1 (200 ng) or MML (50 ng). Each serum sample was diluted 1/100 and tested in duplicate. Bound antibody was detected with protein-A-conjugated horseradish peroxidase. The optimum dilutions for the test sera and conjugates were determined by checker-board titration. The optical density of the wells was read at 405 nm.

#### 2.5.4 Cell isolation and proliferation assay

PBMCs were isolated from heparinized blood samples using standard Ficoll-hypaque gradient centrifugation (Lymphocyte Isolation Solution, RAFER, Spain) and washed twice in 1× PBS. PBMCs at 2.5 × 10^5^ cells/well were cultured in flat-bottomed 96-well plates at 37 °C for 5 days in complete medium (RPMI 1640 supplemented with 100 U/ml penicillin, 100 μg/ml streptomycin, 2 mM l-glutamine, 25 mM HEPES and 10% heat-inactivated foetal calf serum). The cells were incubated in triplicate with either complete media (blank), 10 μg/ml soluble leishmanial antigen (SLA) or 10 μg/ml concanavalin A (ConA). Plates were pulsed during the last 18 h with 1 μCi of methyl-^3^H thymidine and counted in a scintillation counter. Results were expressed as stimulation index (net counts per minute of stimulated cells/net counts per minute of unstimulated cells).

### 2.6 Parasitological analysis

Presence of the parasite was determined by *in vitro* culture and PCR of bone marrow and lymph node aspirates taken every 4 weeks post-challenge. All samples were diluted in 200 μl of PBS.

#### 2.6.1 *In vitro* culture

One hundred microliters of the diluted samples were cultured in di-phasic blood-agar NNN medium, maintained at 27 °C and examined for promastigote forms under light microscopy every week. The original cultures were subcultured into fresh medium every week for up to 4 weeks. The sample was considered negative if there were no parasites observed at the end of this period.

#### 2.6.2 DNA extraction and diagnostic PCR

One hundred microliters of bone marrow, lymph node, skin and PBMC samples were used for DNA extraction. Three hundred microliters of NET10 buffer and 40 μl of 10% SDS were added to each sample, incubated at 70 °C for 1 h and purified using phenol/chloroform extraction and ethanol precipitation. DNA was resuspended in 100 μl of distilled water. *Leishmania* specific nested PCR was performed to detect leishmanial DNA on the different biopsies. Ten microliters of DNA was used as template. In the first amplification 15 pmol of the Kinetoplastida specific primers R221 (GGTTCCTTTCCTGATTTACG) and R332 (GGCCGGTAAAGGCCGAATAG), were mixed with 0.2 mM deoxynucleoside triphosphates (Amersham Pharmacia Biotech, Sweden), 2 mM MgCl_2_, 5 mM KCl, 75 mM Tris–HCl pH 9.0, 2.0 mM (NH_4_)_2_SO_4_, 0.001% bovine serum albumin and 1.4 units of *T*th DNA polymerase (Biotools B&M Laboratories, S.A., Madrid, Spain). The cycling conditions were 94 °C for 5 min followed by 35 cycles of 94 °C for 30 s, 60 °C for 30 s, 72 °C for 30 s, followed by a final extension at 72 °C for 10 min. Samples revealing a 603 bp PCR product were scored as positive for *Leishmania* DNA. Nested PCR (second amplification) was performed using the amplified products from the first reaction together with above mentioned master mix and the *Leishmania-*specific primers (3 pmol each) R223 (TCCCATCGCAACCTCGGTT) and R333 WUGCGGGCGCGGTGCTG′. *T*th DNA polymerase (0.7 units) was added and the annealing temperature raised to 65 °C. Positive samples yielded a PCR product of 358 base pairs [31].

### 2.7 Statistical analysis

Differences between the groups using the mean value ± S.D. was evaluated by Student's *t*-test. Differences over time for a given group were evaluated by Student's *t*-test with matched data.

## 3 Results

### 3.1 Adverse reactions upon vaccination

The different vaccines were well tolerated and only a local reaction (skin inflammation) at the point of inoculation was observed after the second injection in some dogs from groups HASPB1, H1, HASPB1 + H1, and Montanide. Considering that the adjuvant Montanide™ had been included in the preparation of immunizations for all these groups, it was not included in the third inoculation in order to avoid a systemic reaction. Following the third immunization no local reaction was observed in the dogs. One animal from group MML died after the course of immunizations and prior to challenge. Following autopsy examination, the death was found to be related to a gut obstruction and not to the vaccination procedure. The MML group thus had seven animals for the remainder of the study.

### 3.2 Clinical manifestations

**Fig. 1:**
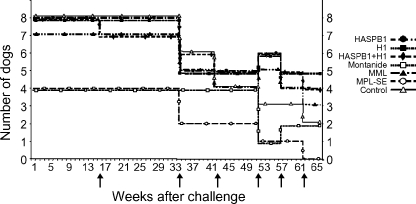
Number of asymptomatic dogs in each group following challenge infection. Clinical examination for symptoms of CVL (lymphoadenopathy, onychogryphosis, alopecia, cutaneous lesions, weight loss or keratoconjunctivitis) was carried out at weeks 16, 34, 42, 52, 57 and 62 post-challenge (indicated by arrows). The initial number of animals for all groups was 8 except for the MML group where the initial number was 7, and the Montanide and MPL-SE groups where the starting number of animals was 4.

Following high dose experimental infection, non-vaccinated control dogs, adjuvant inoculated dogs (groups Montanide and MPL-SE) and antigen plus adjuvant vaccinated dogs (groups HASPB1, H1, HASPB1 + H1 and MML) were periodically checked by an independent experienced clinical veterinarian for the appearance of external clinical manifestations of CVL. The number of dogs in each group that developed patent symptoms of leishmaniasis such as lymphadenopathy, onychogryphosis, alopecia, cutaneous lesions, weight loss and keratoconjunctivitis post-challenge infection is shown in Fig. 1. In the control group, six out of eight animals (75%) developed patent clinical symptoms of leishmaniasis. In adjuvant groups Montanide and MPL-SE, three out of four (75%) and four out of four (100%) animals, respectively, presented clinical manifestations of leishmaniasis. Five out of seven (71%) animals from group MML showed symptoms throughout the trial. In groups HASPB1 and HASPB1 + H1, four out of eight dogs (50%) were symptomatic, and in group H1, only three out of eight (37.5%) animals developed the disease. Symptoms in the dogs appeared from week 32 after experimental infection, except for one dog from group HASPB1 + H1 that showed clinical signs of canine leishmaniasis 16 weeks after challenge.

**Fig. 2:**
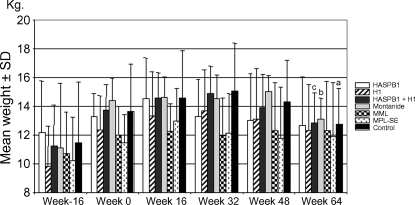
Mean weight of each group of dogs during the study. Data are shown as mean weight ± S.D. in kg. Weights at preimmunization (week −16), postimmunization pre-challenge (week 0), and post-challenge (weeks 16, 32, 48 and 64) are shown. Error bars represent the standard deviation for each group. No statistically significant differences were observed between the groups at the different time points. Statistical analysis using the Student's *t*-test with matched data indicated that there was a decrease of the mean weights for a particular group at the end of the study (week 64) when compared with the mean weight recorded in previous weeks. (a) Significant (*p* < 0.05) decrease of the mean weight in comparison to week 32, (b) significant (*p* < 0.05) decrease of the mean weight in comparison to week 48, and (c) significant (*p* < 0.05) decrease of the mean weight in comparison to week 32 and 48.

Presence or absence of specific symptoms did not correlate with any specific experimental vaccine group and was independent of sex, age and starting weight of the animals. The frequency of clinical symptoms recorded for each group is showed in Table 1. The severity and duration of these clinical manifestations varied between dogs. Some animals presented a progressive polysymptomatic form of the disease, showing multiple and severe symptoms that required, in some cases, that the animal be killed. Other dogs presented few and mild symptoms and remained olygosymptomatic during the course of the study. At each follow up, the weight of the dogs was recorded. Weight loss is a characteristic symptom of CVL. In this study, most of the symptomatic animals lost weight from week 32 until the end of the trial. Decrease of the mean weight between week 32 or 48 and week 64 was observed in groups HASPB1, H1, HASPB1 + H1, Montanide and control, but differences were only significant in dogs from groups HASPB1, Montanide and control (Fig. 2).

### 3.3 Haematology and serum biochemistry

Determination of the haematological values showed that experimental infection induced a progressive decrease in the mean number of total white blood cells, lymphocytes, erythrocytes and platelets (Table 2). Differences in these cell counts between pre-challenge and the end of the dog trial were statistically significant in groups HASPB1, HASPB1 + H1, MML and control. Group H1 showed significant differences only in the case of leucocytes and erythrocytes. Both groups (Montanide and MPL-SE) inoculated with adjuvant only, also showed a decrease in the levels of the different cell counts. Differences between pre-challenge and the last follow up were only significant, however, for platelet (group Montanide) and erythrocyte counts (group MPL-SE). This lack of difference in cell counts for the adjuvant groups is likely due to the small number of animals in these groups.

Serum biochemistry analysis throughout the experiment revealed that serum levels of creatinine and BUN remained within normal levels (0.5–1.8 mg/dl and 7.0–27.0 mg/dl, respectively) in most of the animals (Table 3). Serum levels of alkaline phosphatase (normal range 23–212 U/l) and ALT (normal range 10–100 U/l) were both increased in one symptomatic dog from groups HASPB1, H1, Montanide and MML. Alterations in the serum levels of AST (normal range 0–50 U/l), globulins (normal range 2.5–4.5 g/dl) and total proteins (normal range 5.2–8.2 g/dl) were observed in those animals with multiple patent clinical symptoms of leishmaniasis, independent of the group. Levels of AST were increased over 50 U/l in 84% of the symptomatic animals, while serum globulin levels exceeded the normal range in 92.4% of the symptomatic cases.

### 3.4 Parasite detection

The presence of *L. infantum* parasites was confirmed in bone marrow and lymph node aspirates by culture in NNN biphasic medium and by PCR. Both techniques confirmed the presence of parasites in these target organs from week 4 after challenge. The percentage of *L. infantum*-positive animals in the different groups of dogs throughout the study is shown in Table 4.

For groups HASPB1, Montanide, MML and MPL-SE, the percentage of bone marrow parasite positive animals was similar to that of the control group for the duration of the study. Group HASPB1 + H1 presented higher percentages than the control group, whilst group H1 showed a lower percentage. There were no clear differences in the percentage of animals that were lymph node parasite positive by PCR. In the case of cultured lymph nodes, group MML showed higher percentages than the control group, while group H1 presented a lower proportion of animals that were positive.

### 3.5 Immunological response

**Fig. 3:**
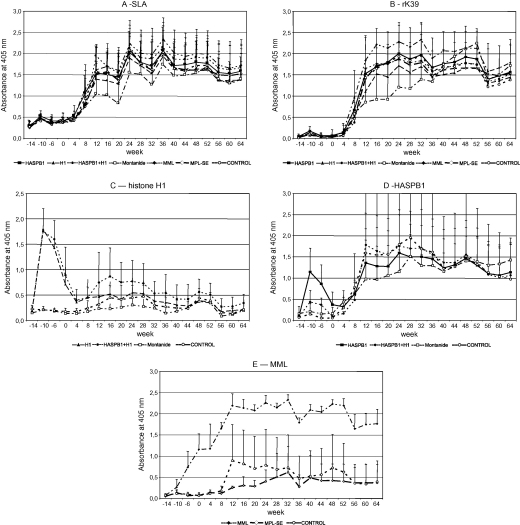
Mean serum levels of specific antibodies. Serum levels of specific antibodies to the antigens SLA (A), rK39 (B), histone H1 (C), HASPB1 (D), and MML (E) were measured during the study by ELISA. Week −14 corresponds to serum levels prior to the immunization process. Weeks −10, −6 and 0, correspond to serum antibody levels after the first, second and third immunization. Week 0 also corresponds to the time of experimental infection. OD readings were measured at 405 nm. Error bars represent the standard deviation for each group.

Specific antibody responses to the antigens used as vaccines was also assessed. Levels of specific serum antibodies to HASPB1, H1 and MML were determined from the pre-immunization stage to the end of the study (Fig. 3). After immunization, total IgG titers against the different antigens were detectable in all antigen immunized groups (HASPB1, H1, HASPB1 + H1 and MML). In the case of groups HASPB1, H1 and HASPB1 + H1, the first immunization induced high levels of specific antibodies against the antigens that decreased after the second and third immunization (Fig. 3C and D). The group vaccinated with the MML antigen, presented a progressive increase in levels of specific antibodies following each immunization (Fig. 3E).

Immunoglobulin titers against crude soluble *leishmania* antigens (SLA) were detectable in all seven groups of dogs from the time of the second follow up. Mean antibody levels for each group increased gradually until weeks 32–36, and then remained high until the end of the study. No significant differences were observed between the mean absorbance of the groups (Fig. 3A). In all experimental groups, symptomatic animals showed the highest levels of anti-SLA serum antibodies while in asymptomatic dogs these levels were moderate. Similar results were obtained for antibodies to the recombinant protein K39 from *L. chagasi* (Fig. 3B).

After challenge, mean antibody titres against histone H1 increased slightly in those groups vaccinated with this antigen (groups H1 and HASPB1 + H1) and around week 24, decreased to remain low until the end of the study. The serum levels of anti-H1 antibodies were higher in those animals immunized with histone H1 that became symptomatic for leishmaniasis than in those that remained asymptomatic. The control group and Montanide adjuvant groups, showed low levels of H1 specific antibodies throughout the experiment (Fig. 3C).

HASPB1 vaccinated groups (HASPB1 and HASPB1 + H1) showed a sharp increase in mean antigen specific antibody levels following challenge and these remained elevated until the end of the study. High titres of anti-HASPB1 antibodies were found in both symptomatic and asymptomatic dogs. This increase in specific antibodies for HASPB1 was also observed in the control and Montanide groups, which confirmed that the effect was due to the experimental infection (Fig. 3D).

Anti-MML titres in the MML group were on average high, and steadily increased after experimental infection to reach a peak level at week 32. No differences were found between the levels of anti-MML antibodies in symptomatic and asymptomatic animals from group MML. The control and MPL-SE groups presented titers 12 weeks post-challenge against MML that were induced by the parasite, but such levels were lower compared to that of group MML (Fig. 3E).

A lymphoproliferative response specific to SLA was not observed in any of the animals following immunization, nor after challenge (Table 5).

## 4 Discussion

Increasing awareness that the dog represents a key target in the control of parasite transmission to humans has promoted interest in development of a vaccine against canine leishmaniasis. This is theoretically feasible, based on the fact that there are a large number of infected dogs in endemic areas amongst whom only a small proportion develop the disease [32,33], together with evidence that naturally infected dogs in endemic areas exhibit lymphoproliferative responses and develop a positive skin test against *Leishmania* antigens [34,35]. Furthermore, recent reports on the immunogenicity and efficacy of several vaccines confirm that protection against CVL is possible.

In the present investigation, we tested vaccines against CVL consisting of the recombinant histone H1 [25,27], HASPB1 [26,36] or MML antigens [22,37]. The histone H1 and HASPB1 antigens were examined individually, or as a cocktail in combination with the adjuvant Montanide™-ISA 720. The MML polyprotein antigen was formulated with the MPL^®^-SE adjuvant. The use of an adequate adjuvant normally constitutes a major aspect in obtaining an efficient protocol of immunization. Montanide™ISA 720 is a mineral oil based adjuvant that has been used in our previous studies in primates [27] and in other trials in dogs [20], without production of severe adverse reactions. In the present study, all dog groups immunized with Montanide™ISA 720 produced local skin reactions. Therefore, a more appropriate adjuvant such as Montanide™-ISA563, which does not contain mineral oil and is easier to inject, may be safer for use in veterinary studies. In the case of HASPB1, protection in mice was achieved in the absence of exogenous adjuvant [26]. However, this unusual attribute of HASPB1 was not tested in the current study. The adjuvant MPL^®^-SE used for the MML vaccine formulation showed no observable adverse reactions in this study nor in previous studies in dogs [22].

Clinical follow up of the animals during the study, together with laboratory analysis allowed the classification of dogs as asymptomatic or symptomatic and hence defined the differential protection capabilities of the vaccine candidates tested. In terms of clinical manifestations, the percentages of animals scored symptomatic by the end of the study were lower in the antigen immunized groups of dogs (ranging from 37 to 70% for groups HASPB1, H1, HASPB1 + H1 and MML), than in control groups immunized with adjuvant only or PBS (from 75 to 100% for groups Montanide, MPL-SE and control). These results indicate that the vaccines containing antigen were able to induce a variable partial protection against *Leishmania* infection, although, due to the low number of animals per group, it was difficult to establish whether such differences were statistically significant. The highest levels of protection were obtained in the group of dogs immunized with histone H1 alone (group B), with 62.5% showing no clinical symptoms. Half (50%) of the animals immunized with HASPB1, either alone or in combination with H1, were protected, and 29% of the animals vaccinated with MML showed no signs of CVL. The vaccine formulation with MML was identical to that used in a previous study in dogs [22], where it was also observed not to confer protection upon natural challenge.

Several physiological, biochemical and haematological alterations such as weight loss, increased serum globulins, anaemia, leucopenia or lymphopenia, are commonly observed in dogs naturally and experimentally infected by *L. infantum* [38]. In this study, we were able to demonstrate that vaccine-induced protection was associated with a reduction of such parameters. An effective vaccine against CVL should allow a normal gain of weight. Significant differences in weight loss between week 32 and the end of the study were only observed in the control group and the group immunized with a mixture of histone H1 and HASPB1 proteins, while significant differences in weight loss between week 48 and the end of the study were observed in the Montanide group. The remainder of the animals did not show significant variations of weight over time. Moreover, experimental challenge was able to induce significant decreases in the levels of leucocytes, lymphocytes, erythrocytes and platelets in most of the groups, although such alterations were not significant in the group of animals immunized with histone H1, which supports the higher percentage of overall protection induced by this antigen. On the other hand, biochemical analysis showed that increased serum component levels are related to the symptomatic conditions of the animal and not to the particular experimental group.

It has been demonstrated that the intensity of tissue parasitism parallels the development of the clinical manifestations of CVL [38–40]. In the case of animals immunized with histone H1, fewer had detectable parasites in the bone marrow and lymph node compared with control group, early after challenge. The other groups of dogs did not show this trend. It was previously indicated that polysymptomatic dogs are more infectious to sand flies than oligosymptomatic dogs [41]. Thus, the reduction of the parasite burden in the canine reservoir through vaccination, even if only partial, as demonstrated here for the histone H1 immunized group, is of interest as it could potentially lead to reduced zoonotic transmission and control of this disease within an endemic area.

Immune recognition of the different recombinant proteins was confirmed by the detection of antigen specific serum antibodies. Serological analysis demonstrated that experimental infection induced a strong humoral response against the parasite antigens SLA and rK39. Interestingly, challenge infection also induced an increase in the serum levels of HASPB1 and MML specific antibodies but not histone H1 specific antibodies. Low anti-histone antibody responses have been observed in other studies [42]. It is claimed that the best predictors of infectiousness for CVL are IgG antibody titres and clinical disease, demonstrated by a positive correlation between anti-*leishmania* IgG, parasite detection by PCR, clinical disease and infectiousness to sand flies [4,38,43,44]. Thus, the low levels of specific antibodies observed in histone H1 immunized animals may be related to their protective capability.

Interestingly, the humoral response against “pathoantigens” which includes cytoplasmic and nuclear parasite antigens such as histones and the proteins that make up the MML vaccine antigen, namely, LmSTII, LeIF and TSA [45], is directly related to the pathological alterations observed in VL patients. Presentation of the intracellular molecules is suggested to occur via cytolysis of amastigotes with the epitopes being localized to a region in each specific sequence that is unique to *Leishmania*, but different or absent in the host homologous protein [45,46].

Recent results in mice indicate that vaccination against *Leishmania* is improved when several distinct antigens are co-administered [23,42,47–51]. However, the results in dogs using multi-component antigens appears to have mixed results with partial protection observed in some instances [18,20] and no protection in others [21,22]. In the present study the cocktail vaccine consisting of HASPB1 and histone H1 antigens or the polyprotein MML vaccine produced a lesser protective effect in dogs than the histone H1 antigen alone.

The high-dose intra-venous challenge infection used in this study proved to be a good method to induce patent CVL in 13 out of 16 control animals (including PBS and adjuvant inoculated animals). However, such strong challenge conditions may have overpowered a protective response present in the vaccinated animals as previously described in other dog vaccine trials [20,21]. When taking into consideration previous attempts of vaccination against CVL, the prevention of severe disease in the majority of animals is clearly difficult to achieve. Dogs are a good model for human visceral leishmaniasis because the symptoms in dogs are similar to those developed in humans [52]. The partial protection obtained in the present study confirms the capacity of recombinant protein vaccination to restrict parasite replication and control infection even under high dose experimental challenge infection. The efficiency of a vaccine against CVL could now be evaluated in natural infection conditions which are likely to be less extreme than those used in this study.

## Figures and Tables

**Table 1:** Frequency of clinical symptoms

Clinical symptoms of CVL	HASPB1	H1	HASPB1 + H1	Montanide	MML	MPL	Control
Lymphadenopathy	37.5	25	50	50	43	50	62.5
Alopecia	37.5	50	62.5	25	71.4	100	87.5
Cutaneous lesions	37.5	25	37.5	25	28.5	75	62.5
Onychogryphosis	50	25	37.5	50	57	50	62.5
Keratoconjuctivitis	37.5	37.5	50	50	43	75	50
Weight loss	37.5	25	25	50	28.5	50	62.5

Values represent the percentage of animals in each group that showed the specific symptom of CVL at week 64.

**Table 2:** Haematological analysis

Group	Haematological values							
	Leucocytes		Lymphocytes		Erythrocytes		Platelets	
	Week 0	Week 64	Week 0	Week 64	Week 0	Week 64	Week 0	Week 64
HASPB1	14.3 ± 3.2	10.0 ± 4.7*	2.10 ± 0.82	1.09 ± 0.45*	7.08 ± 0.37	4.68 ± 1.02*	301 ± 62	203 ± 94*
H1	13.7 ± 2.3	9.7 ± 3.8*	1.80 ± 0.39	1.55 ± 0.82	6.35 ± 0.52	5.01 ± 1.01*	295 ± 97	216 ± 188
HASPB1 + H1	11.3 ± 1.9	6.8 ± 3.3*	2.54 ± 0.52	1.19 ± 0.75*	7.62 ± 1.88	4.27 ± 1.38*	367 ± 51	172 ± 92*
Montanide	12.0 ± 4.4	6.8 ± 3.2	2.02 ± 1.28	0.78 ± 0.32	6.63 ± 0.48	4.51 ± 1.88	274 ± 89	169 ± 41*
MML	11.6 ± 2.6	5.5 ± 2.6*	1.94 ± 0.72	0.78 ± 0.32*	6.94 ± 0.17	3.59 ± 0.92*	353 ± 89	181 ± 80*
MPL-SE	10.5 ± 1.0	8.0 ± 4.0	1.57 ± 0.58	1.8 ± 0.43	7.08 ± 0.43	5.16 ± 1.23*	316 ± 25	244 ± 162
Control	13.5 ± 2.4	7.2 ± 3.5*	2.26 ± 0.69	0.98 ± 0.60*	6.91 ± 0.85	4.21 ± 1.07*	273 ± 66	171 ± 59*

Values are shown for each group at pre-challenge (week 0) and at the end of the study (week 64).

* Statistically significant (*p* < 0.05) differences between the value at pre-challenge (Week 0) and at the end of the trial (Week 64).

**Table 3:** Biochemical analysis

	HASPB1	H1	HASPB1 + H1	Montanide	MML	MPL	Control
AST	37.5	50	75	75	57.1	50	62.5
ALT	12.5	12.5	0	25	14.2	0	0
Alkaline phosphatase	12.5	12.5	0	25	14.2	0	0
BUN	0	12.5	12.5	0	14.2	0	12.5
Creatinine	0	0	0	0	0	0	12.5
Globulins	50	62.5	62.5	75	57.1	75	50
Total proteins	37.5	37.5	37.5	75	14.2	75	12.5

Values represent the percentage of animals in each group that showed serum levels of the different biochemical parameters that exceeded the normal level.

**Table 4:** *In vitro* parasite detection

	Week	HASPB1	H1	HASPB1 + H1	Montanide	MML	MPL-SE	Control
Bone marrow								
Culture	0	0	0	0	0	0	0	0
	16	50	37.5	87.5	50	42.8	75	75
	32	75	50	75	75	57.1	50	75
	48	37.5	25	37.5	50	57.1	50	37.5
	64	37.5	37.5	62.5	100	42.8	50	37.5

PCR	0	0	0	0	0	0	0	0
	16	75	50	87.5	75	57.1	75	75
	32	75	50	75	50	42.8	50	62.5
	48	75	25	75	25	57.1	75	37.5
	64	62.5	37.5	75	75	57.1	50	25

Lymph node								
Culture	0	0	0	0	0	0	0	0
	16	62.5	37.5	75	50	85.7	75	62.5
	32	62.5	62.5	87.5	75	85.7	50	75
	48	75	50	62.5	50	71.4	100	62.5
	64	75	50	37.5	25	71.4	50	62.5

PCR	0	0	0	0	0	0	0	0
	16	62.5	75	75	75	85.7	75	75
	32	50	37.5	50	50	57.1	50	50
	48	25	37.5	50	50	28.5	50	25
	64	62.5	62.5	62.5	50	71.4	75	25

Values represent the percentage of animals in each group that presented positive parasite detection by PCR and culture for bone marrow and lymph node samples prior to the experimental infection (week 0) and at weeks 16, 32, 48 and 64 post-challenge.

**Table 5:** Lymphoproliferative response

	HASPB1	H1	HASPB1 + H1	Montanide	MML	MPL	Control
Week 0							
SLA	1.12 ± 0.8	1.24 ± 0.3	0.97 ± 0.3	1.16 ± 0.4	1.22 ± 0.6	0.98 ± 0.2	1.27 ± 0.4
ConA	7.32 ± 3.6	7.67 ± 2.7	8.17 ± 3.2	8.18 ± 1.8	7.62 ± 2.0	7.42 ± 2.28	8.50 ± 2.8

Week 8							
SLA	0.97 ± 0.1	1.39 ± 0.4	1.00 ± 0.4	1.30 ± 0.3	1.33 ± 0.4	1.18 ± 0.4	1.07 ± 0.33
ConA	6.51 ± 4.0	7.20 ± 2.8	12.12 ± 10.5	14.73 ± 11.7	10.97 ± 9.9	18.0 ± 5.8	10.2 ± 6.1

Week 48							
SLA	1.00 ± 0.3	1.02 ± 0.5	1.32 ± 0.9	1.12 ± 0.39	0.94 ± 0.5	1.13 ± 0.3	1.28 ± 0.2
ConA	2.59 ± 1.0	4.97 ± 2.9	2.88 ± 1.16	6.31 ± 2.3	3.9 ± 1.1	3.81 ± 0.1	3.45 ± 0.9

The results are expressed as the mean stimulation index ± S.D. Lymphoproliferative assays were carried out following immunization (week 0) and at weeks 8 and 48 after experimental infection.

## References

[1] Paranhos-Silva M., Nascimento E.G., Melro M.C., Oliveira G.G., dos Santos W.L., Pontes-de-Carvalho L.C. (1998). Cohort study on canine emigration and *Leishmania* infection in an endemic area for American visceral leishmaniasis. Implications for the disease control. Acta Trop.

[2] Rioux J.A., Lanotte G., Serres E., Pratlong F., Bastien P., Perieres J. (1990). Taxonomy of *Leishmania*. Use of isoenzymes. Suggestions for a new classification. Ann Parasitol Hum Comp.

[3] Baneth G., Dank G., Keren-Kornblatt E., Sekeles E., Adini I., Eisenberger C.L. (1998). Emergence of visceral leishmaniasis in central Israel. Am J Trop Med Hyg.

[4] Courtenay O., Quinnell R.J., Garcez L.M., Shaw J.J., Dye C. (2002). Infectiousness in a cohort of Brazilian dogs: why culling fails to control visceral leishmaniasis in areas of high transmission. J Infect Dis.

[5] Molina R., Amela C., Nieto J., San-Andres M., Gonzalez F., Castillo J.A. (1994). Infectivity of dogs naturally infected with *Leishmania infantum* to colonized *Phlebotomus perniciosus*. Trans Roy Soc Trop Med Hyg.

[6] Alvar J., Molina R., San Andres M., Tesouro M., Nieto J., Vitutia M. (1994). Canine leishmaniasis: clinical, parasitological and entomological follow-up after chemotherapy. Ann Trop Med Parasitol.

[7] Guarga J.L., Moreno J., Lucientes J., Gracia M.J., Peribanez M.A., Castillo J.A. (2002). Evaluation of a specific immunochemotherapy for the treatment of canine visceral leishmaniasis. Vet Immunol Immunopathol.

[8] Gramiccia M., Gradoni L., di Martino L., Romano R., Ercolini D. (1992). Two syntopic zymodemes of *Leishmania infantum* cause human and canine visceral leishmaniasis in the Naples area, Italy. Acta Trop.

[9] Ashford D.A., David J.R., Freire M., David R., Sherlock I., Eulalio M.C. (1998). Studies on control of visceral leishmaniasis: impact of dog control on canine and human visceral leishmaniasis in Jacobina, Bahia, Brazil. Am J Trop Med Hyg.

[10] Dietze R., Barros G.B., Teixeira L., Harris J., Michelson K., Falqueto A. (1997). Effect of eliminating seropositive canines on the transmission of visceral leishmaniasis in Brazil. Clin Infect Dis.

[11] Gradoni L., Gramiccia M., Mancianti F., Pieri S. (1988). Studies on canine leishmaniasis control. 2. Effectiveness of control measures against canine leishmaniasis in the Isle of Elba, Italy. Trans Roy Soc Trop Med Hyg.

[12] Palatnik-de-Sousa C.B., dos Santos W.R., Franca-Silva J.C., da Costa R.T., Reis A.B., Palatnik M. (2001). Impact of canine control on the epidemiology of canine and human visceral leishmaniasis in Brazil. Am J Trop Med Hyg.

[13] Mayrink W., Genaro O., Silva J.C., da Costa R.T., Tafuri W.L., Toledo V.P. (1996). Phase I and II open clinical trials of a vaccine against *Leishmania chagasi* infections in dogs. Mem Inst Oswaldo Cruz.

[14] Gradoni L. (2001). An update on antileishmanial vaccine candidates and prospects for a canine *Leishmania* vaccine. Vet Parasitol.

[15] Borja-Cabrera G.P., Correia Pontes N.N., da Silva V.O., Paraguai de Souza E., Santos W.R., Gomes E.M. (2002). Long lasting protection against canine kala-azar using the FML-QuilA saponin vaccine in an endemic area of Brazil (Sao Goncalo do Amarante, RN). Vaccine.

[16] Nogueira F.S., Moreira M.A., Borja-Cabrera G.P., Santos F.N., Menz I., Parra L.E. (2005). Leishmune vaccine blocks the transmission of canine visceral leishmaniasis: absence of *Leishmania* parasites in blood, skin and lymph nodes of vaccinated exposed dogs. Vaccine.

[17] Lemesre J.L., Holzmuller P., Cavaleyra M., Goncalves R.B., Hottin G., Papierok G. (2005). Protection against experimental visceral leishmaniasis infection in dogs immunized with purified excreted secreted antigens of *Leishmania infantum* promastigotes. Vaccine.

[18] Molano I., Alonso M.G., Miron C., Redondo E., Requena J.M., Soto M. (2003). A *Leishmania infantum* multi-component antigenic protein mixed with live BCG confers protection to dogs experimentally infected with *L. infantum*. Vet Immunol Immunopathol.

[19] Ramiro M.J., Zarate J.J., Hanke T., Rodriguez D., Rodriguez J.R., Esteban M. (2003). Protection in dogs against visceral leishmaniasis caused by *Leishmania infantum* is achieved by immunization with a heterologous prime-boost regime using DNA and vaccinia recombinant vectors expressing LACK. Vaccine.

[20] Rafati S., Nakhaee A., Taheri T., Taslimi Y., Darabi H., Eravani D. (2005). Protective vaccination against experimental canine visceral leishmaniasis using a combination of DNA and protein immunization with cysteine proteinases type I and II of *L. infantum*. Vaccine.

[21] Poot J., Spreeuwenberg K., Sanderson S.J., Schijns V.E., Mottram J.C., Coombs G.H. (2006). Vaccination with a preparation based on recombinant cysteine peptidases and canine IL-12 does not protect dogs from infection with *Leishmania infantum*. Vaccine.

[22] Gradoni L., Foglia Manzillo V., Pagano A., Piantedosi D., De Luna R., Gramiccia M. (2005). Failure of a multi-subunit recombinant leishmanial vaccine (MML) to protect dogs from *Leishmania infantum* infection and to prevent disease progression in infected animals. Vaccine.

[23] Coler R.N., Skeiky Y.A., Bernards K., Greeson K., Carter D., Cornellison C.D. (2002). Immunization with a polyprotein vaccine consisting of the T-Cell antigens thiol-specific antioxidant, Leishmania major stress-inducible protein 1, and *Leishmania* elongation initiation factor protects against leishmaniasis. Infect Immun.

[24] Skeiky Y.A., Coler R.N., Brannon M., Stromberg E., Greeson K., Crane R.T. (2002). Protective efficacy of a tandemly linked, multi-subunit recombinant leishmanial vaccine (Leish-111f) formulated in MPL adjuvant. Vaccine.

[25] Solioz N., Blum-Tirouvanziam U., Jacquet R., Rafati S., Corradin G., Mauel J. (1999). The protective capacities of histone H1 against experimental murine cutaneous leishmaniasis. Vaccine.

[26] Stager S., Smith D.F., Kaye P.M. (2000). Immunization with a recombinant stage-regulated surface protein from *Leishmania donovani* induces protection against visceral leishmaniasis. J Immunol.

[27] Masina S.M.M.G., Demotz S.O., Fasel N.J. (2003). Protection against cutaneous leishmaniasis in outbred vervet monkeys using a recombinant histone H1 antigen. J Infect Dis.

[28] Poot J., Rogers M.E., Bates P.A., Vermeulen A. (2005). Detailed analysis of an experimental challenge model for *Leishmania infantum* (JPC strain) in dogs. Vet Parasitol.

[29] Scott P., Pearce E., Natovitz P., Sher A. (1987). Vaccination against cutaneous leishmaniasis in a murine model. II. Immunologic properties of protective and nonprotective subfractions of soluble promastigote extract. J Immunol.

[30] Burns J.M., Shreffler W.G., Benson D.R., Ghalib H.W., Badaro R., Reed S.G. (1993). Molecular characterization of a kinesin-related antigen of *Leishmania chagasi* that detects specific antibody in African and American visceral leishmaniasis. Proc Natl Acad Sci USA.

[31] Cruz I., Canavate C., Rubio J.M., Morales M.A., Chicharro C., Laguna F. (2002). A nested polymerase chain reaction (Ln-PCR) for diagnosing and monitoring *Leishmania infantum* infection in patients co-infected with human immunodeficiency virus. Trans Roy Soc Trop Med Hyg.

[32] Sideris V., Papadopoulou G., Dotsika E., Karagouni E. (1999). Asymptomatic canine leishmaniasis in Greater Athens area, Greece. Eur J Epidemiol.

[33] Solano-Gallego L., Morell P., Arboix M., Alberola J., Ferrer L. (2001). Prevalence of *Leishmania infantum* infection in dogs living in an area of canine leishmaniasis endemicity using PCR on several tissues and serology. J Clin Microbiol.

[34] Cabral M., O’Grady J., Alexander J. (1992). Demonstration of *Leishmania* specific cell mediated and humoral immunity in asymptomatic dogs. Parasite Immunol.

[35] Cardoso L., Neto F., Sousa J.C., Rodrigues M., Cabral M. (1998). Use of a leishmanin skin test in the detection of canine *Leishmania*-specific cellular immunity. Vet Parasitol.

[36] Alce T.M., Gokool S., McGhie D., Stager S., Smith D.F. (1999). Expression of hydrophilic surface proteins in infective stages of *Leishmania donovani*. Mol Biochem Parasitol.

[37] Fujiwara R.T., Vale A.M., Franca da Silva J.C., da Costa R.T., Quetz Jda S., Martins Filho O.A. (2005). Immunogenicity in dogs of three recombinant antigens (TSA, LeIF and LmSTI1) potential vaccine candidates for canine visceral leishmaniasis. Vet Res.

[38] Reis A.B., Teixeira-Carvalho A., Vale A.M., Marques M.J., Giunchetti R.C., Mayrink W. (2006). Isotype patterns of immunoglobulins: hallmarks for clinical status and tissue parasite density in Brazilian dogs naturally infected by *Leishmania* (*Leishmania*) *chagasi*. Vet Immunol Immunopathol.

[39] Barrouin-Melo S.M., Larangeira D.F., Trigo J., Aguiar P.H., dos-Santos W.L., Pontes-de-Carvalho L. (2004). Comparison between splenic and lymph node aspirations as sampling methods for the parasitological detection of *Leishmania chagasi* infection in dogs. Mem Inst Oswaldo Cruz.

[40] Sanchez M.A., Diaz N.L., Zerpa O., Negron E., Convit J., Tapia F.J. (2004). Organ-specific immunity in canine visceral leishmaniasis: analysis of symptomatic and asymptomatic dogs naturally infected with *Leishmania chagasi*. Am J Trop Med Hyg.

[41] Travi B.L., Tabares C.J., Cadena H., Ferro C., Osorio Y. (2001). Canine visceral leishmaniasis in Colombia: relationship between clinical and parasitologic status and infectivity for sand flies. Am J Trop Med Hyg.

[42] Iborra S., Soto M., Carrion J., Alonso C., Requena J.M. (2004). Vaccination with a plasmid DNA cocktail encoding the nucleosomal histones of *Leishmania* confers protection against murine cutaneous leishmaniosis. Vaccine.

[43] Cabral M., O’Grady J.E., Gomes S., Sousa J.C., Thompson H., Alexander J. (1998). The immunology of canine leishmaniosis: strong evidence for a developing disease spectrum from asymptomatic dogs. Vet Parasitol.

[44] Pinelli E., Rutten V.P., Bruysters M., Moore P.F., Ruitenberg E.J. (1999). Compensation for decreased expression of B7 molecules on *Leishmania infantum*-infected canine macrophages results in restoration of parasite-specific T-cell proliferation and gamma interferon production. Infect Immun.

[45] Requena JM, Alonso C, Soto M. Evolutionarily conserved proteins as prominent immunogens during *Leishmania* infections. Parasitol Today (Personal ed.) 2000;16(6):246–50. 10.1016/s0169-4758(00)01651-3 10827430

[46] Chang K.P., Reed S.G., McGwire B.S., Soong L. (2003). *Leishmania* model for microbial virulence: the relevance of parasite multiplication and pathoantigenicity. Acta Trop.

[47] Mendez S., Gurunathan S., Kamhawi S., Belkaid Y., Moga M.A., Skeiky Y.A. (2001). The potency and durability of DNA- and protein-based vaccines against *Leishmania* major evaluated using low-dose, intradermal challenge. J Immunol.

[48] Parody N., Soto M., Requena J.M., Alonso C. (2004). Adjuvant guided polarization of the immune humoral response against a protective multicomponent antigenic protein (Q) from *Leishmania infantum*. A CpG + Q mix protects Balb/c mice from infection. Parasite Immunol.

[49] Rafati S., Salmanian A.H., Taheri T., Vafa M., Fasel N. (2001). A protective cocktail vaccine against murine cutaneous leishmaniasis with DNA encoding cysteine proteinases of *Leishmania* major. Vaccine.

[50] Rafati S., Zahedifard F., Nazgouee F. (2006). Prime-boost vaccination using cysteine proteinases type I and II of *Leishmania infantum* confers protective immunity in murine visceral leishmaniasis. Vaccine.

[51] Zadeh-Vakili A., Taheri T., Taslimi Y., Doustdari F., Salmanian A.H., Rafati S. (2004). Immunization with the hybrid protein vaccine, consisting of *Leishmania* major cysteine proteinases type I (CPB) and type II (CPA), partially protects against leishmaniasis. Vaccine.

[52] Moreno J., Alvar J. (2002). Canine leishmaniasis: epidemiological risk and the experimental model. Trends Parasitol.

